# Mutated processes predict immune checkpoint inhibitor therapy benefit in metastatic melanoma

**DOI:** 10.1038/s41467-022-32838-4

**Published:** 2022-09-19

**Authors:** Andrew Patterson, Noam Auslander

**Affiliations:** 1 Genomics and Computational Biology Graduate Group, University of Pennsylvania - Perelman School of Medicine, Philadelphia, PA 19104 USA; 2 Program in Molecular and Cellular Oncogenesis, The Wistar Institute, Philadelphia, PA 19104 USA

**Keywords:** Tumour biomarkers, Machine learning, Melanoma

## Abstract

Immune Checkpoint Inhibitor (ICI) therapy has revolutionized treatment for advanced melanoma; however, only a subset of patients benefit from this treatment. Despite considerable efforts, the Tumor Mutation Burden (TMB) is the only FDA-approved biomarker in melanoma. However, the mechanisms underlying TMB association with prolonged ICI survival are not entirely understood and may depend on numerous confounding factors. To identify more interpretable ICI response biomarkers based on tumor mutations, we train classifiers using mutations within distinct biological processes. We evaluate a variety of feature selection and classification methods and identify key mutated biological processes that provide improved predictive capability compared to the TMB. The top mutated processes we identify are leukocyte and T-cell proliferation regulation, which demonstrate stable predictive performance across different data cohorts of melanoma patients treated with ICI. This study provides biologically interpretable genomic predictors of ICI response with substantially improved predictive performance over the TMB.

## Abstract (web summary)

Tumour mutational burden is a biomarker of immune checkpoint inhibitor response, but their association is not fully understood. Here, the authors train classifiers to identify key mutated processes which show stable predictive performance in multiple melanoma cohorts.

## Introduction

Melanoma is a highly aggressive disease and the deadliest form of skin cancer. Deaths from melanoma account for ~60% of skin cancer mortality^1,2^. Prognosis greatly depends on the stage at which the cancer is discovered. Whereas almost all patients diagnosed with localized melanoma survive for at least five years, less than a third of patients diagnosed with distant metastasized melanoma survive over the same period^3^. The majority of patients with metastatic melanoma do not benefit from surgery, chemotherapy and radiation alone^4,5^. Targeted therapies such as BRAF and MEK inhibitors have dramatically improved the prognosis of patients with metastatic melanoma that harbor specific mutations^6–8^. However, only a subset of the patients can benefit from these treatments, and the majority of those develop resistance over time^9,10^. In recent years, Immune Checkpoint Inhibitor (ICI) therapy has been approved for patients with advanced disease, demonstrating durable remission in up to half of the patients^5,9,11^.

The first antibody developed for clinical ICI treatment targets the cytotoxic T-lymphocyte antigen 4 (CTLA-4). CTLA-4 is a T-cell surface protein which binds to B7-1 and B7-2 expressed by antigen-presenting cells (APC)^12^, resulting in suppression of immune response by the T cells. Ipilimumab, a human monoclonal antibody targeting CTLA-4, was the first ICI agent to demonstrate increased progression-free survival (PFS) and overall survival (OS) compared to more traditional cancer treatment methods^12–14^. Subsequently, clinical targeting of the programmed cell death receptor 1 (PD1), which binds to its ligand-receptor PD-L1 to elicit tumor immune escape, has markedly improved the treatment of melanoma and demonstrated durable responses in other types of cancer^15,16^. Several potential new ICI antibodies are currently being explored, such as those targeting the regulatory surface glycoprotein TIM-3^17^. While 40–60% of patients with advanced melanoma experience benefit from ICI, a substantial fraction of patients do not benefit from this treatment, which can incur severe autoimmune adverse events^13,14,18,19^. Therefore, it is critical to uncover tumor characteristics that predict response to ICI.

Numerous biomarkers have been proposed for the prediction of ICI response, but most have not been validated for clinical use. Gene expression biomarkers include PDL-1^20^, CD38^21^, TIM-3^22^, and CXCL9^23^ expression, cytolytic activity^24^, as well as machine learning-derived signatures such as IPRES^25^, TIDE^26^, IMPRES^27^, Immonophenoscores^28^, and others^29,30^. However, a recent meta-analysis evaluated the reproducibility of ICI biomarkers and found that only a subset of these maintained any predictive performance^31^. To date, gene expression signatures predicting ICI response have not been incorporated into clinical use, likely due to limited reproducibility and lack of benchmarking standards, among other factors^32^. Genomic biomarkers of ICI benefit have met more success in terms of clinical use. In 2017, the U.S. Food and Drug Administration (FDA) approved the first biomarker for anti-PD1 efficacy based on high levels of microsatellite instability (MSI-H)^33^. However, MSI-H is only found in a subset of gastrointestinal and endometrial tumors. In 2020, the high tumor mutation burden (TMB-H), quantifying the number of mutations in a tumor, has been approved by the FDA as a marker for anti-PD1 efficacy^34^. While TMB-H has been associated with ICI benefit across different cancer types, there are several challenges for its utility. For example, TMB is tumor type-specific; moreover, TMB-H status does not preclude tumor progression, and low TMB does not preclude response^35,36^. In addition, the mechanism underlying the clinical utility of the TMB is unclear. Therefore, there is a need for additional genomic ICI response biomarkers with improved predictive performance that are more biologically interpretable. Recent studies have examined the mechanistic link between anti-PD1 response or resistance and mutated biological processes such as interferon signaling, MHC presentation, and beta-catenin^37,38^, prompting a need for process-level ICI response biomarkers.

Here, we use tumor mutation data in the context of biological processes to predict patient response to anti-PD1 treatment. We first investigate whether the mutation burden in genes that belong to different biological processes correlate with anti-PD1 benefit. We then apply feature selection methods to distinct processes to identify subsets of genes in which the mutational count predicts anti-PD1 response. This revealed sets of mutated genes in several biological processes with a comparable predictive ability of anti-PD1 response to TMB. Employing nonlinear classification methods further enhanced the predictive performance of classifiers based on mutated genes in specific biological processes. The advantage of these methods is that they can capture intricate relations between the mutated genes in a process and anti-PD1 responses, simultaneously weighing mutations that contribute to either response or resistance. Evaluating decision-tree algorithms and neural network architectures, we found that random forest maintains the most robust performance across different datasets, accurately predicting response and overall survival in independent datasets spanning over 500 melanoma patients in total. In particular, mutations in genes belonging to the leukocyte-proliferation and T-cell regulation processes demonstrate consistently high predictive performances. This study provides a potential way forward for understanding ICI treatment responses and constructing biologically interpretable predictors of treatment benefit based on mutation data.

## Results

### Study design

To evaluate whether mutated genes within biological processes can predict ICI treatment responses in metastatic melanoma, we obtained training and validation mutation and clinical datasets from metastatic melanoma patients treated with anti-PD1. For all experiments, models were trained on the same designated training dataset, and evaluated using the same designated validation dataset (see “Methods”). Throughout this work, we used Gene Ontology (GO)^39,40^ to aggregate genes into biological processes. We first investigated whether the mutation load in genes belonging to distinct biological processes can accurately predict ICI responses. For each GO biological process, we counted the number of mutations in that process per sample in the training datasets and used these values to predict anti-PD1 responses. These analyses revealed that the total mutation counts in distinct biological processes were only mildly predictive of response (Supplementary Data 1). We surmised that only a subset of the mutated genes within specific biological processes may be predictive of ICI responses. To identify subsets of genes within distinct biological processes in which the mutation count best predicts ICI response, we applied feature selection methods to mutations in each biological process.

### Selecting subsets of mutations in biological processes

We used the sum of mutations in selected subsets of genes within distinct biological processes to predict melanoma ICI responders vs. non-responders. The area under the receiver-operating characteristic curve (ROC AUC) was used to evaluate the predictive capacity of mutations in subsets of genes belonging to each biological process. We used a training dataset to build a classification model, and a validation dataset to select biological process-based models with high ICI predictive performance. Both the training and validation datasets are therefore considered part of the training process, in which all biological processes are examined. The subset of biological process-based classifiers that yield substantially better ICI predictive performance compared to the TMB on both the training and validation datasets were later evaluated on independent test datasets, as illustrated in Supplementary Fig. 1A (see “Methods” for information about each dataset). We first employed greedy forward feature selection that iteratively finds the best new feature to add to a set of selected features. In this process, the algorithm starts with an empty set, and then iterates over all genes in a biological process, to add the gene that best improves the predictive performance. When using the greedy forward selected genes within each biological process, several biological processes showed high predictive performance on the training dataset, (ROC AUC >0.75). However, none of these predictors maintained high performance in the validation dataset (that is, at least 90% of the training performance, Supplementary Data 2). We reasoned that the greedy feature selection strategy impaired generalization by converging into local optimum. We therefore applied randomized forward feature selection, which sequentially selects features to add using a probabilistic function (see “Methods” for details). In contrast to the greedy forward selector, four processes that performed well on the training dataset maintained high performance when applied to the validation dataset (Supplementary Data 2 and Supplementary Fig. 1B). These include RNA polymerase II transcription regulation, enzyme regulator activity, the establishment of protein localization, and regulatory regions of nucleic acid binding (Supplementary Fig. 1B). We next applied a genetic algorithm feature selection^41–43^. This method outperformed the forward selection algorithms, where selected subsets of mutated genes in 15 processes maintained high performance on the validation dataset (Supplementary Fig. 1B and Supplementary Data 2). The best-performing processes include immune response, leukocyte differentiation, and cell motility (Supplementary Fig. 1B). Several genes that were frequently selected within these processes have important roles in melanoma progression and prognosis. These include *CD44*, shown to have an effect on tumor progression and subsequent poor prognosis^44,45^ and *TNFSF14*, a regulator of T-cell proliferation that is commonly expressed in melanomas^46^.

Importantly, using all three feature selection methods, the biological processes with best performance on the training dataset performed significantly better on the validation dataset compared to processes that showed poor performance on the training dataset (Supplementary Fig. 1C). We found a positive correlation between the performances of selected subsets of mutated genes in different biological processes across the feature selection methods (Supplementary Fig. 1D). Overall, these results support the premise that subsets of mutated genes within specific biological processes maintain comparable predictive performance to that of the TMB.

### Nonlinear mutational process-based classification

**Fig. 1:**
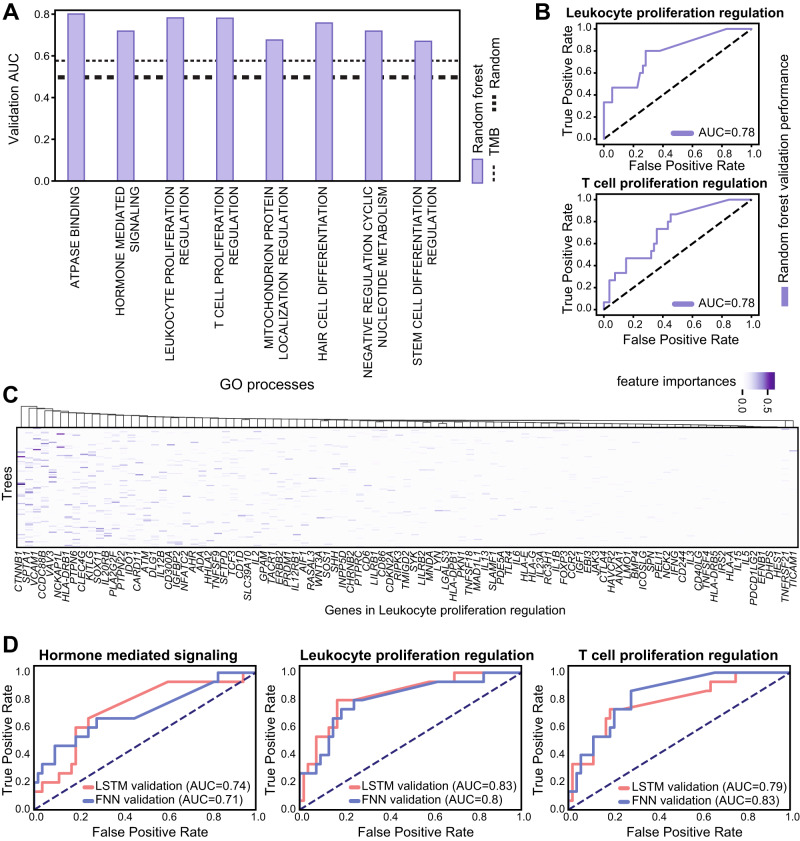
Nonlinear classifiers enhance the prediction performance of melanoma ICI response based on mutations within biological processes. **A** Bar plot showing the RF validation performances for top process-based mutation classifiers. The dashed lines indicate random performance (thick line) and the performance of the tumor mutation burden (thin line). **B** ROC curves demonstrating the RF validation performance when using mutations within leukocyte-proliferation regulation process (upper panel) and within the T-cell proliferation process (lower panel). **C** The genes selected by different trees in the RF model that is trained using mutations in the leukocyte-proliferation regulation process. The values denote feature importance, by mean decrease in impurity. **D** ROC curves demonstrating the validation performances of two neural network architectures (LSTM and FNN) when trained on mutations within the hormone-mediated signaling process (left panel), leukocyte-proliferation regulation process (middle panel), and the T-cell proliferation regulation process (right panel). Source data are provided as Source Data 1.

While using selected subsets of mutated genes indicates several top pathways are approximately equivalent to the TMB, none of the best-performing processes demonstrated a substantial improvement over the TMB. To obtain an ICI response predictor that outperforms the TMB based on tumor mutations, we examined alternative classification techniques. We reasoned that accounting for complex interactions between mutated genes in biological processes may be critical for the prediction of ICI response. We therefore applied nonlinear classifiers to mutated genes within each biological process. First, we trained decision-tree algorithms, including random forest (RF) and gradient boosting (GB), using mutations in all sequenced genes within a biological process. The top biological processes using both methods showed a strong predictive capability across the training and validation datasets (Fig. 1A and Supplementary Fig. 2). In contrast to the sum of mutation classifiers, the top decision-trees predictors substantially exceeded TMB performance for the validation dataset (Fig. 1A, Supplementary Fig. 2, and Supplementary Data 3). Interestingly, leukocyte-proliferation regulation and T-cell proliferation regulation were among the top biological processes, both directly linked to ICI-related immune responses; checkpoint inhibitor antibodies prevent T-cell inhibition and promote the proliferation of effector T cells^47^, and their response to these treatments requires their proliferation and presence in the tumor microenvironment^48^ (Fig. 1B). We investigated the mutated genes in the leukocyte-proliferation regulation process with the highest contribution to the RF prediction capacity. We found that mutations in the beta-catenin gene *CTNNB1* had the highest contribution for prediction, in agreement with recent findings that activation of this gene in melanoma cells is associated with a reduction in T-cell antitumor response^49^. In addition, among the top contributing genes in that process, we found *IL2*, a gene with known antitumor activity by increasing T-cell proliferation and previously used clinically to treat cancers^5,50^, and *CD137*, another known target for antibody-mediated immunotherapy previously tested in clinical trials^51^ (Fig. 1C). To further investigate nonlinear predictors that may capture complex interactions between mutated genes within these processes, we evaluated two classes of neural network models using mutated genes within the top processes. Both the Forward Neural Network and Long Short-Term Memory Recurrent Neural Network models demonstrated high predictive capacity when applied to mutations within these biological processes (Fig. 1D and Supplementary Data 4).

**Fig. 2:**
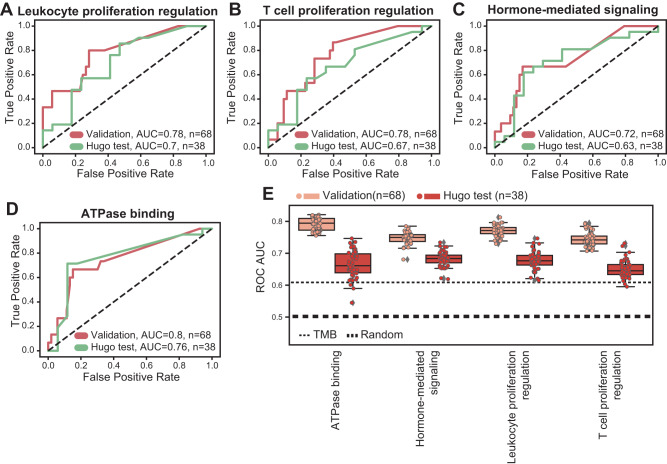
Evaluation of the RF processes classifiers. **A**–**D** ROC curves showing the performances of the RF models for the validation and Hugo test datasets, when trained using mutations within the leukocyte-proliferation regulation process (**A**), the T-cell proliferation regulation process (**B**), the hormone-mediated signaling process (**C**), and the ATPase binding process (**D**). **E** Robustness analysis evaluating the validation performances of the RF process models when retrained with different random seeds. The dashed lines indicate random performance (thick line) and the performance of the tumor mutation burden (thin line). Boxes show the quartiles (0.25 and 0.75) of the data, center lines show the medians, and whiskers show the rest of the distribution except for outliers. Source data are provided as Source Data 2.

To test the potential clinical utility of the selected biological process-based predictors, we examined their performance using an additional test dataset where not all genes used for training were sequenced. This dataset^25^ comprises mutation and response data from 38 melanoma patients treated with anti-PD1, but included only 59–68% of the genes used to train the classifiers (Supplementary Data 5). This data was unseen for the complete training and validation process, and only the selected classifiers that demonstrated high predictive performance in the validation dataset were evaluated in this dataset. Remarkably, despite this, the process mutation RF classifiers maintained their high predictive performance for this dataset (Fig. 2A–D and Supplementary Fig. 3). To test the robustness of this approach we evaluated these classifiers when retrained using different random seeds (see “Methods”). This analysis revealed that the performance on both unseen datasets is maintained with the RF classifiers and is consistently better compared to TMB (Fig. 2E). Notably, RF classifiers were the most robust when presented with missing features in the test dataset^25^ (Supplementary Fig. 3). Importantly, we found only mild correlations between the overall TMB and the classification scores yielded by the RF predictors, supporting that these biological process-based classifiers are capturing more than just an estimate of the TMB (Supplementary Fig. 4). Moreover, using a bootstrapping process, we find that the top RF classifiers perform significantly better than the TMB (Supplementary Fig. 5A, B). As expected, the number of genes in a process strongly correlates with the RF predictor performance in the training dataset (by allowing more complex decision rules), however, there is only slight association between the number of process genes and predictor performance in the validation dataset (Supplementary Fig. 5C). Further exploring this, we found that using different classifier thresholds, more responding patients are correctly predicted with the leukocyte-proliferation regulation RF predictor compared to the TMB (Supplementary Fig. 6). As a result, some responding patients that are not captured by the TMB are predicted as responders by the leukocyte-proliferation regulation RF classifier (Supplementary Data 6).

**Fig. 3:**
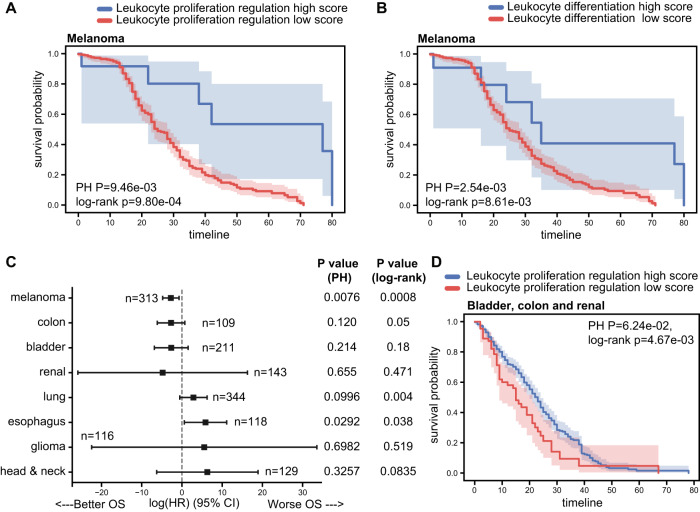
Mutations in leukocyte-proliferation and differentiation processes predict anti-PD1 overall survival. **A** Kaplan–Meier survival curves comparing between anti-PD1 treated melanoma patients with high vs low-prediction scores of the RF model, when trained using mutations within the leukocyte-proliferation regulation process. The Cox proportional hazards as well as the log-rank *P* values are indicated. **B** Kaplan–Meier survival curves comparing anti-PD1-treated melanoma patients with high vs low number of selected mutations in the leukocyte differentiation process, using the genetic algorithm feature selection. The Cox proportional hazards as well as the log-rank *P* values are indicated. **C** The coefficient (bar centers) of the proportional hazards RF predictor using mutations within the leukocyte-proliferation regulation process, for different cancer types. Error bands show the standard errors of the coefficients. **D** Kaplan–Meier survival curves comparing anti-PD1-treated patients with high- vs low-prediction scores of the RF model, when trained using mutations within the leukocyte-proliferation regulation process, using an integrated data of bladder, colon and renal cancer. For Kaplan–Meier curves, shaded areas represent the confidence interval of survival. The Cox proportional hazards as well as the log-rank *P* values are indicated. Source data are provided as Source Data 3.

To further evaluate the potential clinical utility of these classifiers, we assessed their ability to predict overall survival in an independent dataset, the Memorial Sloan Kettering Cancer Center (MSKCC) data of patients treated with anti-PD1^52^. These data were also kept unseen for the training and validation process and were used to test only the selected classifiers that demonstrated high predictive performance in the validation. This MSKCC dataset includes 321 melanoma and skin cancer patients treated with anti-PD1, of which 313 had clinical follow-up data. This mutation data is limited to only 468 genes in the MSK-IMPACT targeted set. Nevertheless, the four RF mutated process models trained previously were significantly predictive of survival in this dataset, and in particular, the leukocyte-proliferation regulation process was significant and strongly predictive (Fig. 3A and Supplementary Fig. 7). Using the predictors based on sum of mutations and the genetic algorithm feature selection, we found that higher number of mutations in the leukocyte differentiation process was predictive of ICI response (Supplementary Fig. 1B). We found that the sum of mutations in selected genes in this process was also strongly predictive of overall survival in the MSKCC dataset (Fig. 3B). To evaluate the performance of the leukocyte-proliferation regulation RF classifier in another treatment context, we applied the model, without further training, to predict response to CTLA-4 inhibitor therapy through an independent dataset^53^. Even though it was trained to predict anti-PD1 response, the leukocyte-proliferation regulation RF classifier was predictive of anti-CTLA-4 response, demonstrating potential utility in a larger clinical context (Supplementary Fig. 8).

### Pan-cancer-mutated pathway outcome prediction

We then evaluated whether the leukocyte-proliferation regulation RF classifier, which obtained the best performance over all datasets, may be applicable to other cancer types. To this end, we applied it to predict overall survival for other cancer types included in the MSKCC dataset. In addition to melanoma, three cancers (colon, bladder, and renal) showed a positive association between the leukocyte-proliferation regulation predictor and overall survival following anti-PD1 treatment (Fig. 3C). When pooling samples from the three non-melanoma cancer types together, the leukocyte-proliferation regulation predictor demonstrated significant overall survival predictive capability via log-rank test (Fig. 3D).

**Fig. 4:**
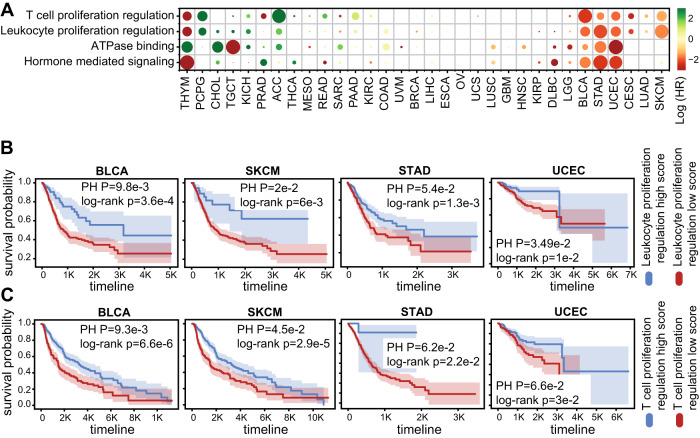
Biological process-based random forest classifiers predict overall survival in TCGA. **A** Heatmap showing the hazard ratios (log-transformed) obtained through overall survival prediction using the classification scores of the selected process-based random forest classifiers. **B** Kaplan–Meier survival curves comparing between TCGA samples from four cancer types, with high vs low-prediction scores of the RF model, when trained using mutations within the leukocyte-proliferation regulation process. The Cox proportional hazards and log-rank *P* values are indicated. **C** Kaplan–Meier survival curves comparing between TCGA samples from four cancer types, with high vs low-prediction scores of the RF model, when trained using mutations within the T-cell proliferation regulation process. For Kaplan–Meier curves, shaded areas represent the confidence interval of survival. The Cox proportional hazards as well as the log-rank *P* values are indicated. Source data are provided as Source Data 4.

Finally, we evaluated the prognostic value of the top RF predictors derived through this work in different cancer types from The Cancer Genome Atlas (TCGA) dataset. To this end, we applied the classifiers that were trained on the Liu data based on mutations within the four selected biological processes to 32 cancer types from TCGA. We found that the leukocyte and T-cell proliferation regulation process RF classifiers were predictive of overall survival in SKCM, UCEC, STAD, and BLCA (Fig. 4A–C). Importantly, for the latter three cancer types, all four RF process classifiers were significantly predictive of overall survival. The leukocyte-proliferation regulation RF classifier was the strongest predictor of survival across TCGA cancer types. Our analysis in Fig. 1C showed that beta-catenin gene, *CTNNB1*, contributes most to classification in the leukocyte-proliferation regulation RF model. While *CTNNB1* activation has been associated with immune exclusion in melanoma cells^49^, it may be associated with improved ICI responses on T cells. To better understand the context in which *CTNNB1* contributes to the prediction of ICI response, we applied CIBERSORT^54^ to TCGA samples, and investigated the association between *CTNNB1* mutations and the predicted abundances of different immune cell types. Interestingly, we found that different subsets of CIBERSORT-inferred T cells are significantly higher in *CTNNB1* mutated melanoma tumors compared to wild-type *CTNNB1* tumors (Supplementary Fig. 9 and Supplementary Data 7). To better understand the association between the leukocyte-proliferation regulation RF classifier with ICI response in different cancer types, we correlated the classifier scores with mutation signatures^55^ in different cancer types through TCGA (Supplementary Fig. 10 and Supplementary Data 8). We found that in SKCM, the strongest correlation observed was with signature 7, which is linked with ultraviolet light exposure. Similarly, we found the strongest correlation in LUAD to be signature 4, linked with tobacco smoking, and the strongest association with COAD to be signature 6, linked with defective mismatch repair^56^.

## Discussion

Understanding the mechanisms underlying response and resistance to ICI therapy is critical to improving treatment of melanoma as well as other types of cancer. Through different feature selection and classification methods, we have shown that analyzing tumor mutations in the context of biological processes enhances the predictive performance of ICI response compared to existing genomic predictors. Using feature selection methods, we identified subsets of genes within distinct biological processes in which the mutation burden presents an alternative biomarker to the genome-wide TMB. To further enhance the predictive performance, we trained nonlinear classifiers using mutated genes in distinct biological processes. We reasoned that nonlinear classification methods have the potential to capture complex associations between ICI responses and mutated genes within a process. We found that using a random forest method substantially improves the predictive capability of predictors trained using mutations in specific processes, demonstrating significantly better performance compared to the TMB. Among the processes that maintain the best performance are leukocyte and T-cell proliferation regulation, known to play an important role in immune infiltration and ICI treatment. The predictive performance of these process classifiers is consistent across multiple datasets, and remain stable across varying sequencing coverage.

We investigate different methods to predict treatment benefit using mutations in the context of biological processes, which demonstrate several notable improvements over the TMB. First, the models in this work require substantially fewer genes to be sequenced for prediction. For example, the leukocyte-proliferation regulation predictor requires sequencing of 99 genes, and the T-cell proliferation regulation predictor requires sequencing of 73 genes. We further investigated whether using a smaller subset of genes within these processes would retain a similar predictive power. We found that less than 20 genes were sufficient to maintain a comparable performance, with the caveat that for this analysis, we evaluated the performance on the three datasets together (Supplementary Data 9). Second, developing biomarkers based on distinct biological processes improves their interpretability, and allows investigation of the mechanisms underlining their clinical utility. In particular, we found that using nonlinear classifiers substantially improves the predictive capability of mutated processes, by simultaneously accounting for mutations associated with either resistance or response to treatment. The methods implemented throughout this work may be applied to construct mutated process predictors of response to other treatments in different cancer types, as evidenced by the prognostic value demonstrated in the TCGA analysis.

More generally, we found that somatic mutations within distinct immune and signaling processes have a strong predictive performance of ICI responses in melanoma. This finding suggests that interactions between tumor genetic alterations and the microenvironment underlie, at least in part, ICI responses. This could be facilitated through altered antigen presentation, supported by several HLA mutations that are frequently selected in trees within the random forest classifier (Fig. 1C). Alternatively, or in complement, it is possible that mutated signaling processes modulate immune infiltration in the tumor microenvironment, supported by the selection of mutations in multiple signaling genes such as beta-catenin and protein kinase and phosphatase genes (Figs. 1B and 2C). Supporting this notion, we found that beta-catenin mutations are associated with increased CIBERSORT-inferred abundances of different T-cell subsets (Supplementary Fig. 9). Interestingly, we find only moderate correlation between the leukocyte-proliferation regulation classifier scores with B- and T-cell burden scores (BCB and TCB, respectively) that have been published recently^57^, supporting an independent prognostic value (Supplementary Fig. 11). In addition, patients with high BCB or TCB scores are not associated with increased response, as reported^57^, whereas patients with high leukocyte-proliferation regulation classifier scores are associated with response, supporting the potential clinical value of this classifier (Supplementary Fig. 11).

We additionally found that different processes were identified when using the mutation count classifiers than those identified with nonlinear classification methods. Interestingly, the leukocyte differentiation process was selected using the genetic algorithm feature selection, whereas the leukocyte-proliferation regulation was selected using the decision-tree algorithms. It is possible that while mutated leukocyte differentiation process is associated with ICI response, some of the mutated genes in the leukocyte-proliferation regulation process may be associated with ICI resistance. Importantly, genes belonging to the leukocyte-proliferation regulation process but not in the leukocyte differentiation process include several MHC class I complex genes (*HLA-A*, *E, G, DRB1, DRB5*, and *DPB1*), which are known to be associated with immune evasion and ICI resistance^58,59^.

This study also has several potential limitations that are important to discuss. First, despite the improved predictive performance of random forest classifiers, RF and similar methods are more complex and often less interpretable for clinical use. Nevertheless, this is not the first study demonstrating that nonlinear classification methods can significantly improve the prediction of ICI benefit^60^. Incorporating clinical features to train random forest models may potentially further improve the performance obtained in this work, when data becomes available^60^. In addition, future developments may dissect the biological processes distinguished in this work to identify candidate targets to enhance treatment sensitivity. Second, similar to the TMB, the predictive models developed in this study account only for tumor factors and not for the tumor microenvironment. Third, it remains open to investigation whether the biological processes distinguished throughout this work for melanoma also determine ICI response in other types of cancer.

In conclusion, this study investigates mutated biological processes that predict ICI response by employing different machine learning methods, and pinpoints specific processes that are highly predictive of ICI benefit in melanoma. If further investigated and validated using additional data cohorts, the predictors developed throughout this work may present a compelling alternative to the tumor mutation burden for predicting patient response to ICI therapy.

## Methods

### Datasets

For training, we used 144 melanoma patients’ samples from ref. ^61^, including somatic mutations and anti-PD1 response information. For validation, we used 68 melanoma patients’ samples with somatic mutations and clinical data from ref. ^62^. To further test the models, we used 38 anti-PD1-treated melanoma patients’ samples from ref. ^25^. For all datasets, responders were defined as patients with complete or partial response. We additionally utilized targeted mutation data and overall survival data from the MSKCC cohort^52^, including melanoma, colorectal, bladder, renal, lung, esophagus, glioma and head and neck cancers. CTLA-4 data is from 110 metastatic melanoma patients from ref. ^53^.

TCGA mutation data were downloaded from the Xena Browser^63^ (https://xenabrowser.net).

The processing of the WES cohorts is described in the original publication^21,51,52^. Briefly, these were processed using MuTect and Strelka for identification of small insertions or deletions. Generalization of a classifier to different cohorts across different processing methods is crucial to support its potential clinical utility. For further evaluation of the datasets, we provide the sex and age distributions across the cohorts (whenever available) in Supplementary Fig. 12.

### Feature selection for biological processes mutation load predictors

We applied three feature selection methods to mutations in genes belonging to each biological process, to select a subset of genes that best predict ICI response. To this end, the predictive performance is defined to be the resulting ROC AUC when using the number of mutations in selected genes in a process as scores, and the ICI response as labels. The following feature selection methods were applied to the training dataset:

1. Greedy Forward Selector. The greedy forward selection algorithm iteratively selects genes within a process that improves the predictive performance. The algorithm starts with an empty list of genes, and at each step, it adds to that list the gene (in a specific biological process) that results in the highest performance when added. For each biological process, we ran a maximum of ten iterations, where the stopping criteria was when ten iterations were completed, or when none of the genes in a process improved the performance when added.
2. Probabilistic Forward Selector. The probabilistic forward selector algorithm is similar to the greedy forward selector, except that the selection of the gene to add in each step is randomized over a set of possible genes. We defined a probability to add a gene that improves the performance when added to be $\frac{1}{{{{{{\rm{number}}}}}}\,{{{{{\rm{of}}}}}}\,{{{{{\rm{total}}}}}}\,{{{{{\rm{iterations}}}}}}+{{{{{\rm{current}}}}}}\,{{{{{\rm{iteration}}}}}}}$
3. Genetic Algorithm. The following steps of the Genetic Algorithm were applied to each biological process (a) Initialization of a population of size 20, where approximately 10% of the genes in the biological process were randomly selected for each instance in the initial population. (b) Evaluation of each instance in the population, where mutations in each gene set in the population were summed to predict ICI response. (c) The top half of the instances in the population, that is, those with the best predictive performance, were selected for reproduction, with randomly selected pairing. (d) Crossover was applied to the randomly selected pairs, until a population size of 20 was reached. Ten iterations of steps (b−d) were repeated, and the best solution was retained, corresponding to the sets of mutated genes that yielded the best performance predicting ICI response.

### Decision-tree predictors for mutations within different biological processes

We trained decision trees to predict ICI response using the training dataset, where the classification scores obtained with these predictors were used to predict ICI response. The following algorithms were considered:

1. Random forest. Random Forest generates multiple decision trees from subsets of features of the data, which are ensembled into a single classifier, therefore reducing the risk of overfitting for large decision trees. We used RandomForestClassifier method from the sklearn.ensemble package, with 100 estimators, a max depth of 5 and a minimum sample split of 2. Other parameters were defined to default.
2. Gradient boosting. Gradient uses boosting to integrate relatively shallow decision trees and ensemble a set of weak learners into a single strong learner. We used GradientBoostingClassifier method from the sklearn.ensemble package, with 100 estimators, a max depth of 2, a learning rate of 0.1, and the deviance loss function. All other parameters defined to default.

For reproducibility, the random state was set to 100 throughout this work, except for the robustness analysis.

When testing on datasets with missing values (where some of the genes were not sequenced) the decision-tree classifiers were retrained on the training dataset with the original random seed, for the subset of genes present in the new data.

### Neural network predictors for mutations within different biological processes

We additionally trained two neural network architectures to predict ICI response, where the resulting classification scores were used for prediction. These include:

1. Feed Forward Neural Network, using one fully connected hidden layer with five hidden units and sigmoid activation.
2. Long Short-Term Memory (LSTM) recurrent neural networks, using one LSTM cell with five hidden units.

All neural networks were trained with tensorflow.keras, using Adam optimizer, with 100 epochs and a batch size of 27.

### Robustness analysis

To evaluate the robustness of different methods, we retrained the classifiers using the mutations within the selected processes and evaluated the performance of 50 retrained classifiers for each selected process.

### Survival analysis

Survival analysis was performed using the proportional hazards, using python lifelines.statistics package. Either the sum of mutations per process (genetic algorithm and forward feature selection) or the classification scores (decision trees and neural networks) were used for prediction. We evaluated all results when controlling for age and sex as confounders and stratified for different cancer types in analyses aggregating patients with different cancer types.

### Bootstrapping analysis

To evaluate the significance at which the random forest classifiers outperform the TMB in predicting ICI response, based on the four processes selected in training, we performed a bootstrap analysis. We downsampled 75% of each cohort 1000 times, applied each of the four top RF classifiers to the downsampled cohort, as well as the TMB, to obtain the prediction AUCs. The fraction of AUCs from the downsampling procedure in which the TMB outperformed the RF classifiers were used as a permutation *P* value.

### Downsampling analysis

To evaluate the smallest subsets of genes that retain the predictive capability of the full set of genes in a process, we randomly subsampled genes from each of the four processes previously selected in training. For each run, 15–85% of the genes were subsampled and used to train an RF model for each pathway. This was run 10,000 times for each pathway to determine the smallest subsets of genes which still retained predictive power across the datasets from Liu, Riaz, and Hugo comparable to the previously generated models (>0.7 ROC Score).

### Statistics and reproducibility

Data was divided into training, validation, and test sets, which corresponded to data from ref. ^61^, ref. ^62^, and ref. ^25^, respectively. To minimize potential overfitting and improve the generalizability of classifiers in new datasets, training, validation, and testing datasets were used in full. No data was excluded from any dataset. Models were trained on the training dataset and validated using the validation dataset. The first author was blinded to the test dataset during training and validation. The four pathways that performed significantly better than the TMB were tested on the test dataset. While small variations in performance are always expected when using different random seeds, the results are robust for random seed selection and maintain significantly improved performance compared to the TMB (Fig. 2E and Supplementary Fig. 3).

### Reporting summary

Further information on research design is available in the Nature Research Reporting Summary linked to this article.

### Supplementary information

Supplementary information

Reporting Summary

Supplementary Dataset 1

Supplementary Dataset 2

Supplementary Dataset 3

Supplementary Dataset 4

Supplementary Dataset 5

Supplementary Dataset 6

Supplementary Dataset 7

Supplementary Dataset 8

Supplementary Dataset 9

Supplementary Dataset 10

### Source data

Source Data

## Data Availability

All data associated with this study are publicly available and additionally provided through the github directory [https://github.com/AuslanderLab/Mutated_pathway_ICI_prediction] and Zenodo^64^ [https://zenodo.org/record/6998939]. The Liu et al.^61^ training dataset, the Van Allen et al.^53^ data, and data from the MSKCC cohort^52^ were downloaded from cBioPortal^65^ [https://www.cbioportal.org]. The Riaz et al.^62^ validation dataset and Hugo et al.^25^ test dataset were obtained through supplementary information of the respective publications. TCGA mutation data was downloaded from the Xena Browser^63^ [https://xenabrowser.net]. The mutated biological process-based prediction scores generated in this study are provided as Supplementary Data 10.  Source data are provided with this paper.

## Code availability

The code to implement and reproduce all analyses presented in this work is provided through the GitHub directory [https://github.com/AuslanderLab/Mutated_pathway_ICI_prediction]. The sample code has been deposited to Zenodo^64^ at https://zenodo.org/record/6998939.
