# Author Correction: Mutated processes predict immune checkpoint inhibitor therapy benefit in metastatic melanoma

**DOI:** 10.1038/s41467-023-41662-3

**Published:** 2023-09-21

**Authors:** Andrew Patterson, Noam Auslander

**Affiliations:** 1 Genomics and Computational Biology Graduate Group, University of Pennsylvania - Perelman School of Medicine, Philadelphia, PA 19104 USA; 2 Program in Molecular and Cellular Oncogenesis, The Wistar Institute, Philadelphia, PA 19104 USA

**Keywords:** Tumour biomarkers, Machine learning, Melanoma

Correction to: *Nature Communications* 10.1038/s41467-022-32838-4, published online 19 September 2022

The original version of this article contained an error in Fig. 2E, in which the labels on the x-axis of “Leukocyte proliferation regulation” and “T cell proliferation regulation” were inadvertently swapped. This has been corrected in both the PDF and HTML versions of the article.

The original version of the Supplementary Information associated with this article contained an error in Supplementary Fig. 3B, in which the labels on the x-axis of “Leukocyte proliferation regulation” and “T cell proliferation regulation” were inadvertently swapped. The HTML has been updated to include a corrected version of the Supplementary Information.

### Supplementary information

Updated Supplementary Information

