# Supplementary material for: Mutated processes predict immune checkpoint inhibitor therapy benefit in metastatic melanoma
Source: Nat Commun. 2022 Sep 19;13:5151. doi: 10.1038/s41467-022-32838-4 (PMC9485158; doi:10.1038/s41467-022-32838-4)
Supplement: Supplementary file 1 — Supplementary information [file 41467_2022_32838_MOESM1_ESM.pdf]

Supplementary Figures

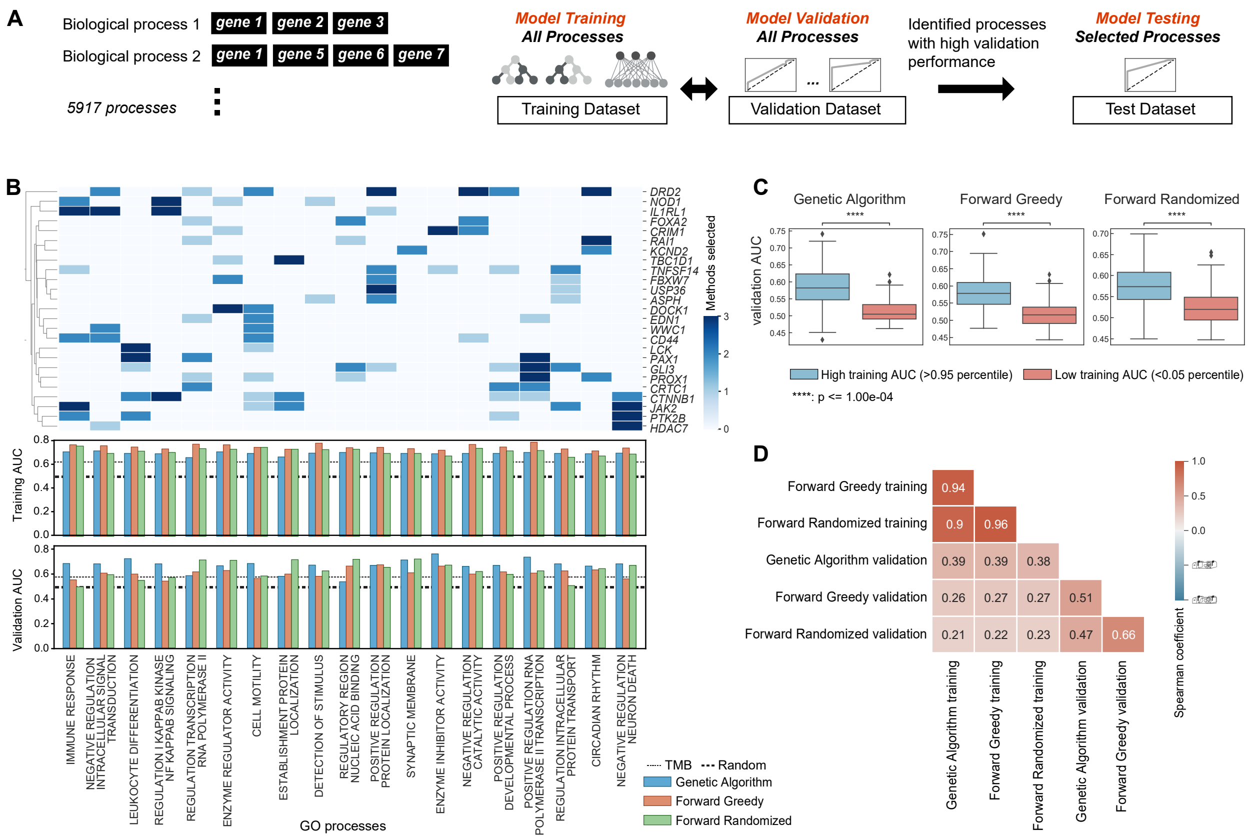

Supplementary Figure 1. Subsets of mutated genes within biological processes predict melanoma ICI response. (A) Schematic overview of the training, validation and testing process throughout this study. Mutations within each biological process were considered and used to train a classifier on the training datasets, then the performance of each classifier was evaluated through the validation dataset. Only a small subset of biological process based classifiers with high performance on the validation datasets were further considered and applied to the test dataset. **(B)** Bar plot comparing the performances of different feature selection methods for the training dataset (middle panel) and the validation dataset (lower panel). The upper panel shows the genes that are most frequently selected within these processes. The dashed lines indicate random performance (thick line) and the performance of the tumor mutation burden (thin line). **(C)** Boxplot comparing the validation performance between the processes that performed best and worst on the training dataset (top and bottom 5%), using the three feature selection methods. **(D)** Correlation heatmap showing the Spearman's correlation coefficient when correlating performances (AUC) between the training and validation datasets using the three feature selection methods.

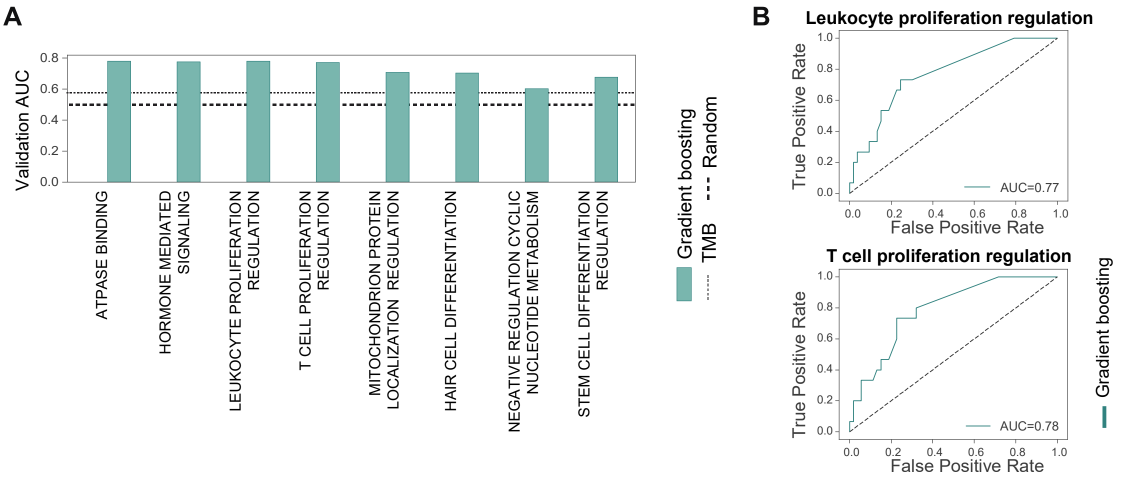

Supplementary Figure 2. (A) Bar plot showing the GB validation performances for top pathway based mutation classifiers. The dashed lines indicate random performance (thick line) and the performance of the tumor mutation burden (thin line). **(B)** ROC curves demonstrating the GB validation performance when using mutations within leukocyte proliferation regulation process (upper panel) and within the T-cell proliferation process (lower Panel).

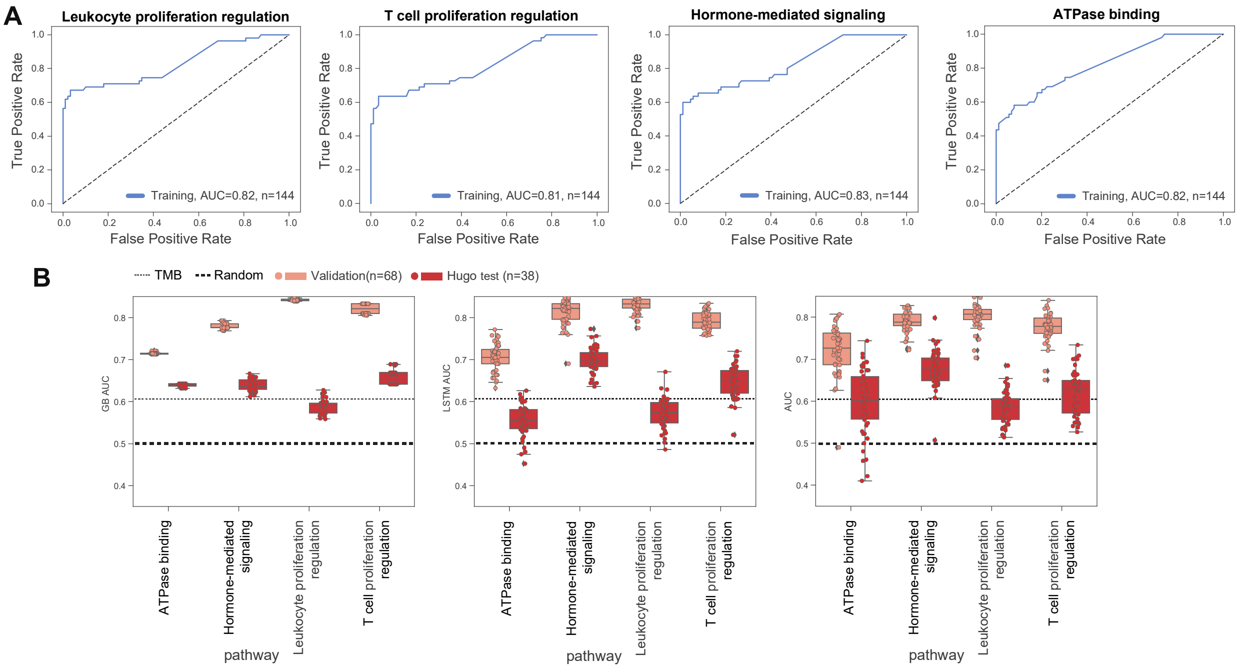

Supplementary Figure 3. (A) ROC curves showing the performances of the RF models for the training dataset. **(B)** Robustness analysis evaluating the validation performances of GB, LSTM, and FNN pathway models when retrained with different random seeds. The legends indicating the four processes have been corrected, where the following processes were swapped in the original version: leukocyte proliferation regulation, T cell proliferation regulation. The dashed

lines indicate random performance (thick line) and the performance of the tumor mutation burden (thin line).

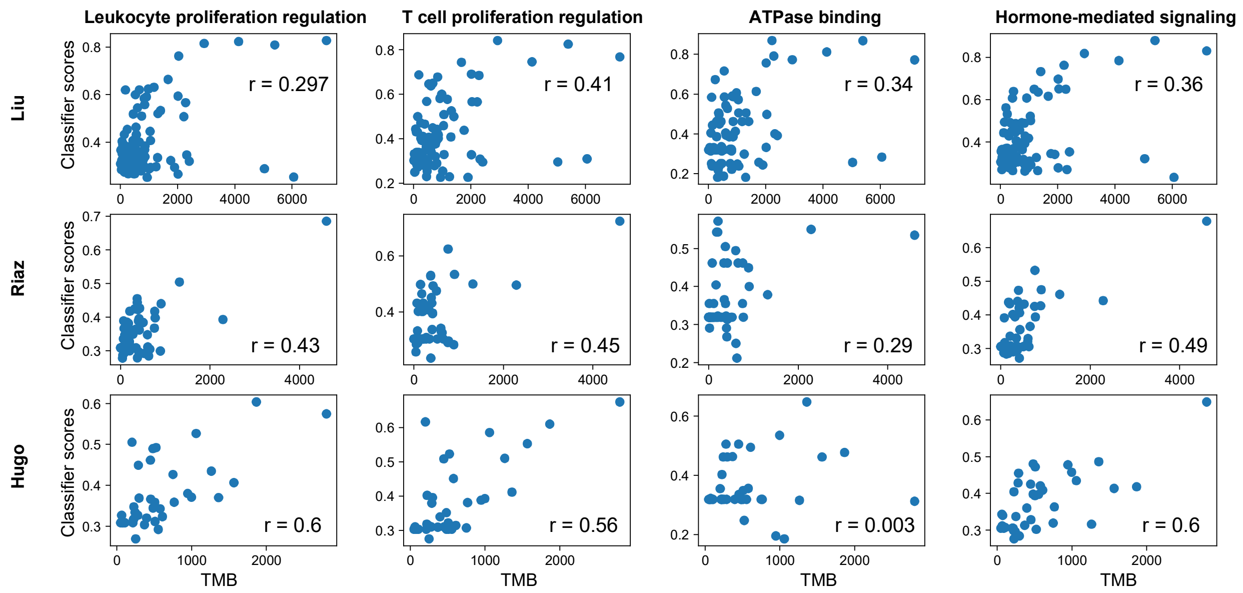

Supplementary Figure 4. Scatter plots correlating the prediction scores of the top four RF classifiers that were selected in training (y-axes) to the TMB (x-axes) in the training (Liu), validation (Riaz) and test (Hugo) datasets. The Spearman correlation coefficients are provided.

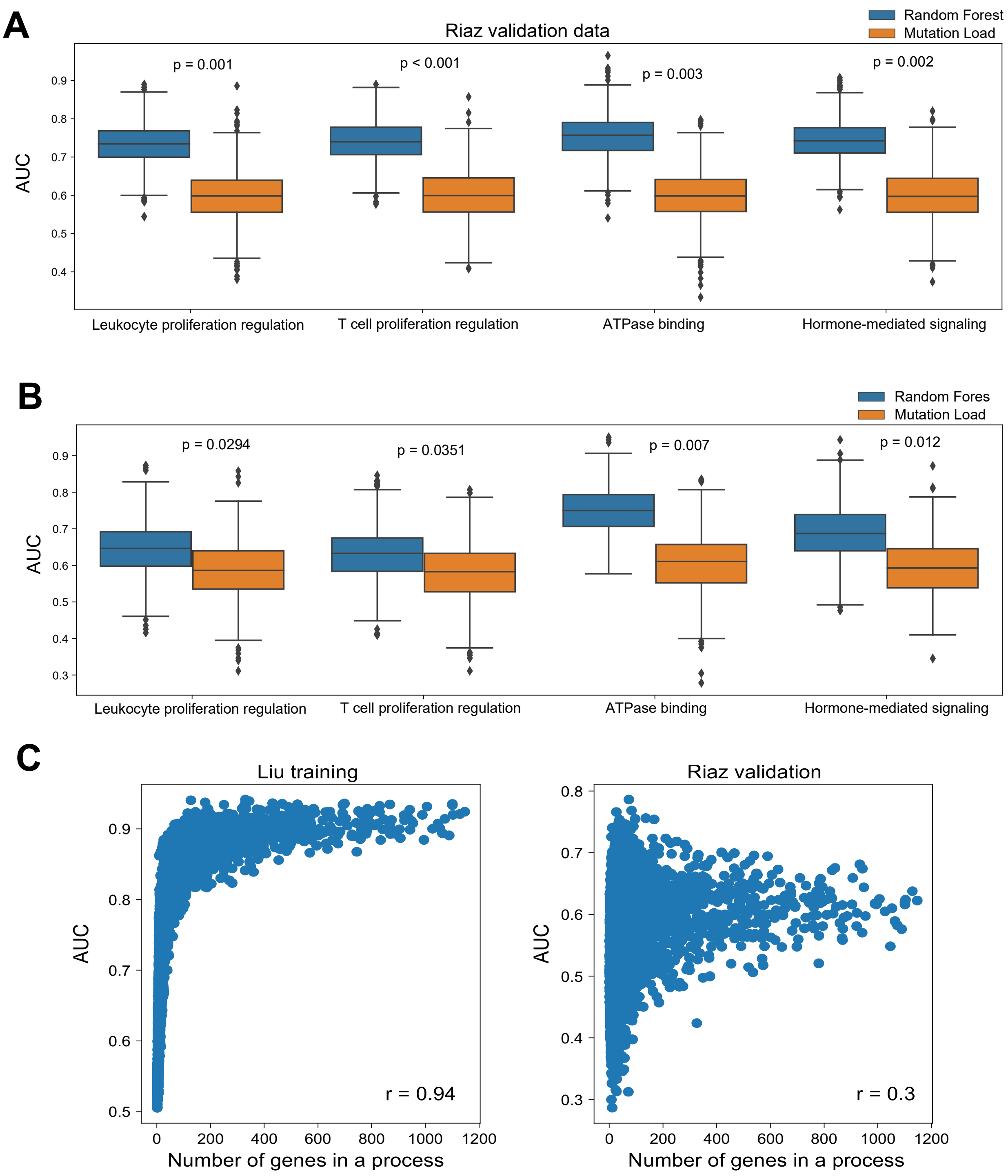

Supplementary Figure 5. Comparison of the performances of the four top RF classifiers that were selected in training to that of the TMB through bootstrapping, for the validation dataset **(A)** and the test dataset **(B)**. **(C)** Scatter plots correlating the model AUC with the number of genes in a biological process in the training (left panel) and validation (right panel) datasets. The Spearman correlation coefficients are provided. As evident, while the correlation is substantial in the training dataset, there is weak correlation in the validation dataset.

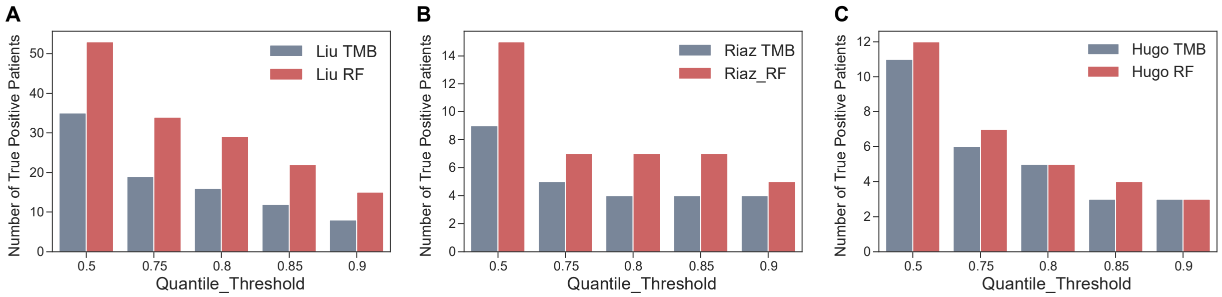

Supplementary Figure 6. The number of responding patients that are correctly predicted as responders through the leukocyte proliferation regulation process RF predictor in **(A)** Liu training dataset, **(B)** Riaz validation dataset and **(C)** Hugo test dataset.

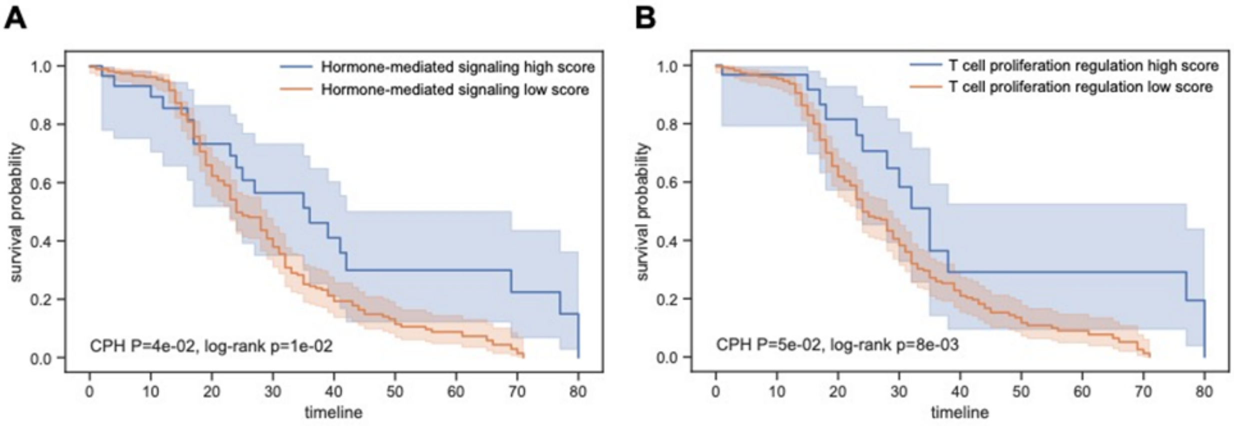

Supplementary Figure 7. Kaplan Meier survival curves comparing between anti-PD1 treated melanoma patients with high vs low prediction scores of the RF model, when trained using mutations within the hormone-mediated signaling process **(A)** and the T-cell proliferation regulation process **(B)**.

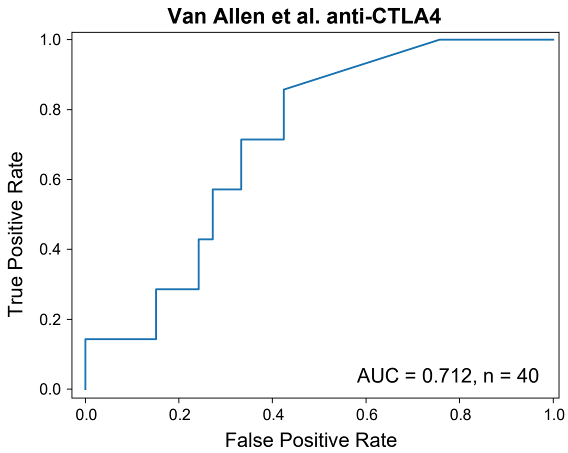

Supplementary Figure 8. ROC curve demonstrating the performance of the leukocyte proliferation regulation RF classifier when applied to the Van Allen et al. melanoma anti-CTLA4 dataset.

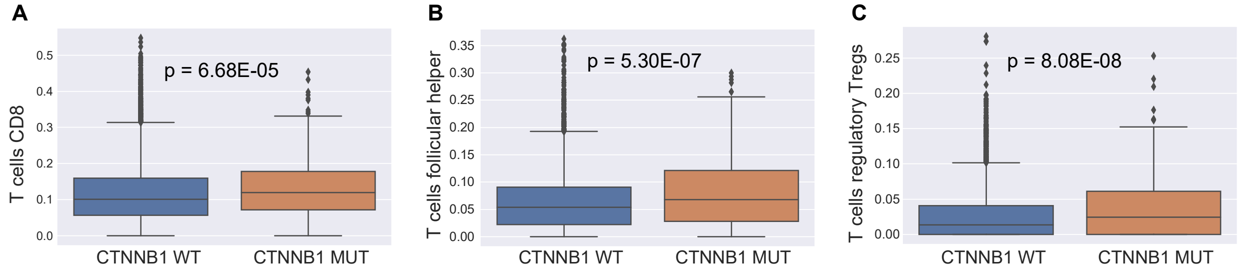

Supplementary Figure 9. Boxplots comparing CIBERSORT inferred CD8 T cells **(A)** follicular helper T cells **(B)** and T regs **(C)**, between CTNNB1 wild-type (WT) and mutated (MUT) tumors.

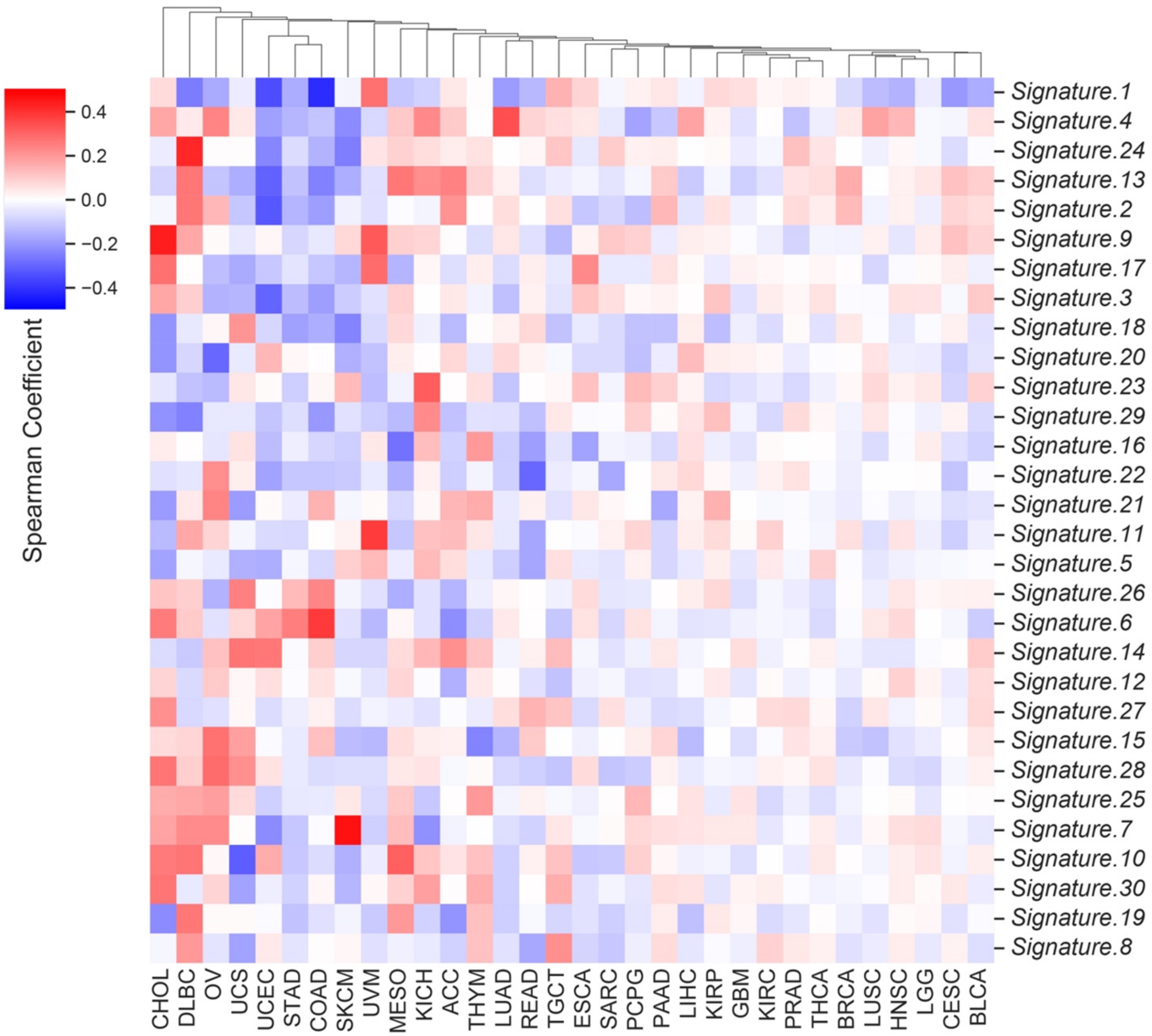

Supplementary Figure 10. Correlation heatmap plot showing the Spearman correlation coefficient correlating the leukocyte proliferation RF classifier scores with mutation signatures (y-axis) in different cancer types (x-axis).

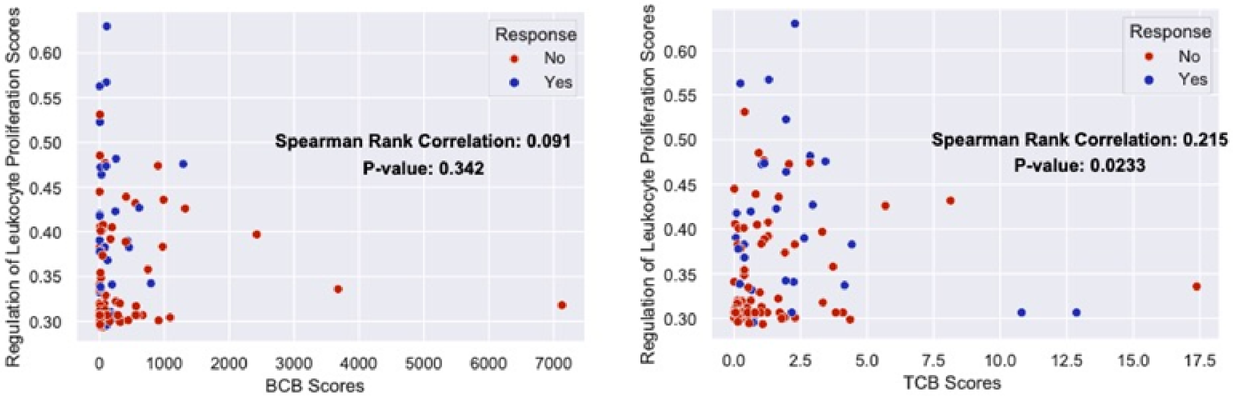

Supplementary Figure 11. Scatter plots correlating the BCB and TCB scores (x-axes) with the leukocyte proliferation regulation classifier scores (y-axes) for responder (blue) and non-responder patients (red).

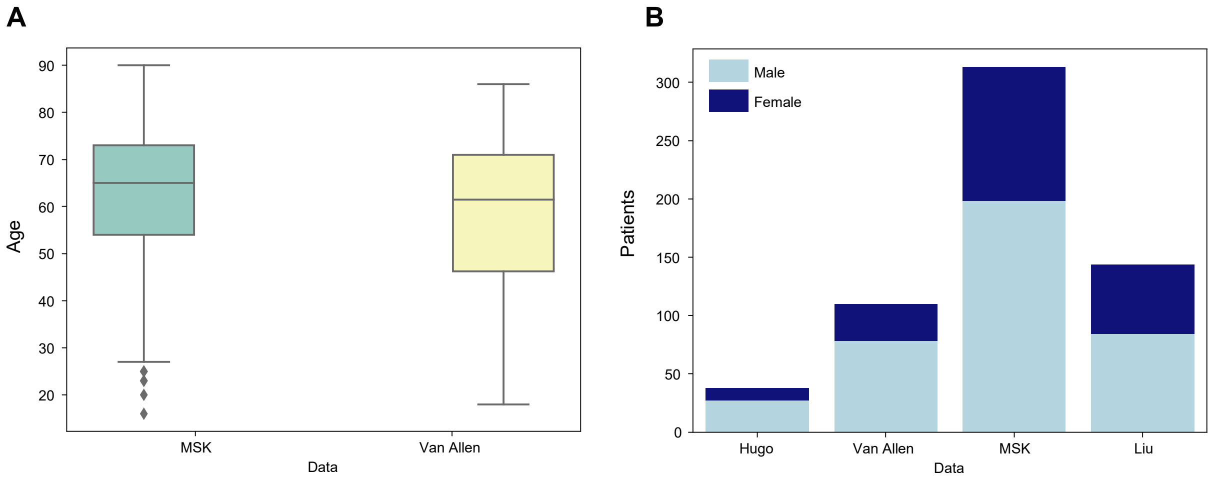

Supplementary Figure 12. (A) Boxplots comparing the age distribution between the MSK cohort and the Van Allen anti-CTLA4 cohorts, for which age data is available. **(B)** Bar plots showing the number of males vs. females in samples in the cohorts where sex is available.

Supplementary Tables

Supplementary Table 1. ROC AUC values of total mutations load in each biological process (Gene Ontology processes) for the training (Liu) and validation (Riaz) datasets.

Supplementary Table 2. ROC AUC values for each biological process when using feature selection methods: Greedy Forward Selection, Probabilistic Forward Selection, and Genetic Algorithms. Column GO_processes: Gene Ontology Biological Processes. Column auc_liu_GO_FS_response: ROC AUC values for the process after Greedy Forward Selection on the training dataset. Column auc_riaz_GO_FS_response: ROC AUC values for the process after Greedy Forward Selection on the validation dataset. Column

auc_liu_GO_FS_response_randomized: ROC AUC values for the process after Probabilistic Forward Selection on the training dataset. Column auc_riaz_GO_FS_response_randomized: ROC AUC values for the process after Probabilistic Forward Selection on the validation dataset. Column auc_liu_GO_GA_response: ROC AUC values for the process after the Genetic Algorithm on the training dataset. Column auc_riaz_GO_GA_response: ROC AUC values for the process after the Genetic Algorithm on the validation dataset.

Supplementary Table 3. ROC AUC values for each process using the Random Forest and Gradient Boosting decision tree algorithms for the training and validation datasets. The first column are the pathways (biological processes), the second column (train_RF_AUC) are the AUC values of the random forest on the training dataset, the third column (validation_RF_AUC) are the AUC values of the random forest on the validation dataset, the fourth column (train_GB_AUC) are the AUC values of the gradient boosting algorithm on the training dataset, and the fifth column (validation_GB_AUC) are the AUC values of the gradient boosting algorithm on the validation dataset.

Supplementary Table 4. ROC AUC values for select pathways using the Forward Neural Network (FNN) and Long Short-Term Memory neural network (LSTM) algorithms. The first column are the GO processes, the second, third and fourth column are the FNN AUC values for the training, validation, and test datasets, respectively. The fifth, sixth, and seventh columns are the LSTM AUC values for the training, validation, and test datasets, respectively.

Supplementary Table 5. The number of genes in a pathway after intersection between the indicated datasets, or the number of genes in a single dataset. The first column indicates the intersection, and the rest of the columns are the pathways.

Supplementary Table 6. The patients that have low TMB and high classifier scores (through the RF leukocyte proliferation regulation predictor), using the median and the 0.75 of the classifier or TMB scores, for the training, validation and test datasets.

Supplementary Table 7. The Wilcoxon rank-sum p-values comparing the CIBERSORT inferred abundances between CTNBB1 mutated TCGA SKCM samples and CTNBB1 wild-type TCGA SKCM samples.

Supplementary Table 8. The Spearman correlation coefficient and p-values correlating the mutation signatures (columns) with the leukocyte proliferation regulation RF classifier scores, when applied to different cancer types from TCGA (rows).

Supplementary Table 9. Feature reduction analysis applied to the top four biological process RF classifiers, including the ROC AUC for the training, validation and test, as well as the genes selected in each iteration.
