# Supplementary material for: Mutated processes predict immune checkpoint inhibitor therapy benefit in metastatic melanoma
Source: Nat Commun. 2022 Sep 19;13:5151. doi: 10.1038/s41467-022-32838-4 (PMC9485158; doi:10.1038/s41467-022-32838-4)
Supplement: Supplementary file 2 — Reporting Summary [file 41467_2022_32838_MOESM2_ESM.pdf]

nature portfolio

Corresponding author(s): Noam Auslander

Last updated by author(s): 08/05/2022

Reporting Summary

Nature Portfolio wishes to improve the reproducibility of the work that we publish. This form provides structure for consistency and transparency in reporting. For further information on Nature Portfolio policies, see our Editorial Policies and the Editorial Policy Checklist.

Statistics

For all statistical analyses, confirm that the following items are present in the figure legend, table legend, main text, or Methods section.

n/a	Confirmed	
☐	☒	The exact sample size (*n*) for each experimental group/condition, given as a discrete number and unit of measurement
☐	☒	A statement on whether measurements were taken from distinct samples or whether the same sample was measured repeatedly
☐	☒	The statistical test(s) used AND whether they are one- or two-sided<br>*Only common tests should be described solely by name; describe more complex techniques in the Methods section.*
☐	☒	A description of all covariates tested
☐	☒	A description of any assumptions or corrections, such as tests of normality and adjustment for multiple comparisons
☐	☒	A full description of the statistical parameters including central tendency (e.g. means) or other basic estimates (e.g. regression coefficient) AND variation (e.g. standard deviation) or associated estimates of uncertainty (e.g. confidence intervals)
☐	☒	For null hypothesis testing, the test statistic (e.g. *F*, *t*, *r*) with confidence intervals, effect sizes, degrees of freedom and *P* value noted<br>*Give P values as exact values whenever suitable.*
☐	☒	For Bayesian analysis, information on the choice of priors and Markov chain Monte Carlo settings
☐	☒	For hierarchical and complex designs, identification of the appropriate level for tests and full reporting of outcomes
☐	☒	Estimates of effect sizes (e.g. Cohen's *d*, Pearson's *r*), indicating how they were calculated

Our web collection on statistics for biologists contains articles on many of the points above.

Software and code

Policy information about availability of computer code

Data collection	All data was collected from public repositories.<br>Liu et al. and the MSK datasets were downloaded from cBioPortal: http://www.cbioportal.org<br>Riaz et al., Van Allen and Hugo et al. datasets were downloaded through the respective publications<br>TCGA mutation data was downloaded from the Xena Browser (https://xenabrowser.net)<br><br>Data handling was carried out in python using pandas version 1.3.3 through 1.4.3
Data analysis	All classifiers were trained using the training datasets, and applied to the validation datasets to select biological process based classifiers with consistent high performance. The selected process classifiers were then applied to left out test datasets.<br><br>Statistical tests were carried out using the scipy.stats python module version 1.7.0 through 1.8.0. Ensemble classifier methods and model performance calculations used scikit-learn version 1.0.0 through 1.1.0. Neural Networks used Keras version 2.8.0. Survival Analysis was carried out using lifelines version 0.26.3 through 0.27.1.<br><br>All data and code associated with this study are publicly available and additionally provided through the github directory: https://github.com/AuslanderLab/Mutated_pathway_ICI_prediction

For manuscripts utilizing custom algorithms or software that are central to the research but not yet described in published literature, software must be made available to editors and reviewers. We strongly encourage code deposition in a community repository (e.g. GitHub). See the Nature Portfolio guidelines for submitting code & software for further information.

Data

Policy information about availability of data

All manuscripts must include a data availability statement. This statement should provide the following information, where applicable:

- Accession codes, unique identifiers, or web links for publicly available datasets
- A description of any restrictions on data availability
- For clinical datasets or third party data, please ensure that the statement adheres to our policy

All data associated with this study are publicly available and additionally provided through the github directory: https://github.com/AuslanderLab/Mutated_pathway_ICI_prediction
The mutated biological process based prediction scores generated in this study are provided as Supplementary Data 10

Field-specific reporting

Please select the one below that is the best fit for your research. If you are not sure, read the appropriate sections before making your selection.

☒ Life sciences ☐ Behavioural & social sciences ☐ Ecological, evolutionary & environmental sciences

For a reference copy of the document with all sections, see nature.com/documents/nr-reporting-summary-flat.pdf

Life sciences study design

All studies must disclose on these points even when the disclosure is negative.

Sample size	All samples from each study was included
Data exclusions	No data was excluded from analysis
Replication	The performances were replicated 50 times using 50 different random seeds for training the decision tree classifiers
Randomization	To reduce overfitting and show robust generalizability, training, validation, and testing datasets were used as provided and patients were not randomized between datasets. No data was excluded from the training, validation, or testing datasets.
Blinding	The test datasets were kept unseen until final models were selected and established

Reporting for specific materials, systems and methods

We require information from authors about some types of materials, experimental systems and methods used in many studies. Here, indicate whether each material, system or method listed is relevant to your study. If you are not sure if a list item applies to your research, read the appropriate section before selecting a response.

Materials & experimental systems

n/a	Involved in the study	
☒	☐	Antibodies
☒	☐	Eukaryotic cell lines
☒	☐	Palaeontology and archaeology
☒	☐	Animals and other organisms
☒	☐	Human research participants
☒	☐	Clinical data
☒	☐	Dual use research of concern

Methods

n/a	Involved in the study	
☒	☐	ChIP-seq
☒	☐	Flow cytometry
☒	☐	MRI-based neuroimaging
